# Gender differences in murine pulmonary responses elicited by cellulose nanocrystals

**DOI:** 10.1186/s12989-016-0140-x

**Published:** 2016-06-08

**Authors:** Anna A. Shvedova, Elena R. Kisin, Naveena Yanamala, Mariana T. Farcas, Autumn L. Menas, Andrew Williams, Philip M. Fournier, Jeffrey S. Reynolds, Dmitriy W. Gutkin, Alexander Star, Richard S. Reiner, Sabina Halappanavar, Valerian E. Kagan

**Affiliations:** 1 Health Effects Laboratory Division, NIOSH, Exposure Assessment Branch, 1095 Willowdale Road, Morgantown, WV 26505 USA; 2 Engineering and Controls Technology Branch, NIOSH/CDC, Morgantown, WV USA; 3 Department of Physiology & Pharmacology, School of Medicine/WVU, Morgantown, WV USA; 4 Environmental and Radiation Health Sciences Directorate, Health Canada, Ottawa, ON K1A 0 K9 Canada; 5 Department of Chemistry, University of Pittsburgh, Pittsburgh, PA USA; 6 Department of Pathology, University of Pittsburgh, Pittsburgh, PA USA; 7 Forest Products Laboratory, USDA Forest Service, Madison, WI USA; 8 Free Radical Center, University of Pittsburgh, Pittsburgh, PA USA; 9 Department of Environmental & Occupational Health, University of Pittsburgh, Pittsburgh, PA USA

**Keywords:** Cellulose, Gender differences, Pulmonary toxicity, Inflammation, Oxidative stress, mRNA expression

## Abstract

**Background:**

Cellulose-based materials have been used for centuries to manufacture different goods derived from forestry and agricultural sources. In the growing field of nanocellulose applications, its uniquely engineered properties are instrumental for inventive products coming to competitive markets. Due to their high aspect ratio and stiffness, it is speculated that cellulose nanocrystals (CNC) may cause similar pulmonary toxicity as carbon nanotubes and asbestos, thus posing a potential negative impact on public health and the environment.

**Methods:**

The present study was undertaken to investigate the pulmonary outcomes induced by repeated exposure to respirable CNC. C57BL/6 female and male mice were exposed by pharyngeal aspiration to CNC (40 μg/mouse) 2 times a week for 3 weeks. Several biochemical endpoints and pathophysiological outcomes along with gene expression changes were evaluated and compared in the lungs of male and female mice.

**Results:**

Exposure to respirable CNC caused pulmonary inflammation and damage, induced oxidative stress, elevated TGF-β and collagen levels in lung, and impaired pulmonary functions. Notably, these effects were markedly more pronounced in females compared to male mice. Moreover, sex differences in responses to pulmonary exposure to CNC were also detected at the level of global mRNA expression as well as in inflammatory cytokine/chemokine activity.

**Conclusions:**

Overall, our results indicate that there are considerable differences in responses to respirable CNC based on gender with a higher pulmonary toxicity observed in female mice.

**Electronic supplementary material:**

The online version of this article (doi:10.1186/s12989-016-0140-x) contains supplementary material, which is available to authorized users.

## Background

Cellulose-based materials have been used for centuries to manufacture a number of different goods derived from forestry and agricultural sources (wood, hemp, cotton, flax plants, etc.). In the increasingly growing field of cellulose applications, their uniquely engineered properties (e.g. mechanical, thermal, rheological and optical) are instrumental for inventive products coming to competitive market. High consumer demand, industrial growth, and government regulation/funding challenges the production of contemporary goods derived from sustainable and renewable sources. These technologically advanced materials must also meet safety standards of low environmental impact and negative health effects to animals/humans [1].

Cellulose is the most abundant organic constituent produced by plants and tunicates and is the major structural component in plant cells and tissues. Cellulose is a long chain natural polymer that plays an essential role in the human food cycle. Cellulose has many applications in industry including building insulation, veterinary foods, additive in plastics/coatings, wood and paper, fibers and clothes, cosmetics and pharmaceutical industries [2]. Nanocellulose (NC) is produced from a variety of cellulose sources, either by hydrolysis [3, 4] or oxidation [5], giving rise to nanocrystalline structures with different dimensions and surface chemistries. Cellulose nanowhiskers or nanocrystals (CNC) are a crystalline form of NC produced by acid hydrolysis of cellulose fibers employing either sulfuric or hydrochloric acid. CNC are rod-like, highly crystalline particles (relative crystallinity index above 75 %) with a rectangular cross section. Their dimensions depend on the intrinsic cellulose source, hydrolysis and temperature. CNC used in this study are produced by the treatment of wood pulp with 64 % sulfuric acid. This hydrolyzes the amorphous entity of the cellulose polymer yielding an acid resistant CNC product. Due to their unique physical/chemical characteristics, many other potential applications exist for CNC including a variety of coatings, packaging, rheological modifiers, and reinforcing agents in nanocomposites. CNCs may also be utilized in the production of detergents, transparent materials for the military (including a wide range of defense-related applications, lightweight transparent armor, eye protection and face shields), multiphase compositions to stabilize aqueous paint mixtures, personal care products (shampoos, detergents, conditioners), and as texturing agent in food industry (ice-creams and whip cream substitutes).

It has been previously reported that pulmonary exposure of rats to cellulose fibers (CF) caused hyperplasia and mild interstitial fibrosis of alveolar cells. Low soluble CF are not efficiently cleared from the animal lungs thus causing long bio-persistent effects [6–10]. In workers exposed to cellulose, there was a higher prevalence of respiratory symptoms and asthmatic episodes, with a decrease in pulmonary function [11–14]. Ericsson et al (1988) have reported an increase in the prevalence of upper respiratory tract outcomes that are dose dependent, while Thorén et al, (1989, 1994) described an increased risk for asthma and chronic obstructive pulmonary diseases in workers from paper mills. Due to the high frequency of respiratory symptoms, a reduction of exposure to cellulose dust was recommended [15]. As of today, there are limited studies available documenting pulmonary toxicity of cellulose based nanomaterials, in particular CNCs. We have previously reported that short term exposure of C57BL/6 mice to respirable CNC caused pulmonary damage, accelerated oxidative stress, acute pulmonary inflammation with accelerated recruitment of macrophages, neutrophils, lymphocytes and eosinophils found in bronchial alveolar lavage fluids (BAL) 24 h post treatment [16].

Gender dissimilarities in airway responses, the lung’s mechanical properties, and the clinical manifestations of pulmonary disease are observed throughout the human life span and are related to biological - as well as sociocultural – factors [17–23]. Sex differences in the incidence and prognosis of a number of respiratory diseases have been reported [17]. It has been shown that women are at an increased risk of adverse health outcomes elicited by air pollution and smoking compared to men [18, 19]. Higher rates of maladies and mortality was also noted in females exposed to ozone and other gaseous pollutants-leading to lung inflammation/injury, loss of immune function, and an increased risk of respiratory infection [22, 23].

The present study was undertaken to investigate the pulmonary outcomes induced by repeated exposure to respirable CNC. C57BL/6 female and male mice were treated by pharyngeal aspiration with CNC (40 μg/mouse) 2 times a week for 3 weeks. The primary goal of this study was to determine whether gender affects pulmonary function, global mRNA expression, and cytokine/chemokine inflammatory responses in the lung of C57BL/6 mice. This was achieved by comparison of pulmonary function, pathology outcomes, lung injury and inflammatory responses, oxidative stress bio-markers, gene ontology, pathway analysis, prediction of upstream regulatory transcription factors in lungs of male and female mice. The results presented herein clearly indicate considerable gender differences in response to respirable CNC with a higher rate of pulmonary toxicity observed in female mice.

## Methods

### CNC characterization

CNC solutions were prepared by suspending the as-received solid samples in water. Atomic force microscopy (AFM) samples were prepared by drop casting 5 μL of 20 mg/L sample solution on freshly cleaved mica. AFM analysis was conducted using a Multimode scanning probe microscope (Veeco Instruments Inc., Waltham MA) in tapping mode. An ACL probe (AppNano, Mountain View, CA) was utilized at a frequency between 160–225 kHz, an amplitude set point between 1.5–1.8 V, and a drive amplitude between 100–300 mV. The resulting images were processed using Gwyddion (Brno Czech Republic). Dynamic light scattering analysis (DLS) was performed using a Brookhaven Instrument Corporation ZetaPALS (Holtsville, NY). 10 runs were averaged using an assumed refractive index of 1.474 (real). Scanning electron microscopy (SEM) samples were prepared by drop casting 5 μL of 2 g/L sample solution on a silicon wafer that had been treated with piranha solution (7:3 concentrated H_2_SO_4_:30 % H_2_O_2_ at 70 °C) for 30 min immediately before sample deposition. SEM and energy dispersive X-ray spectroscopy (EDX) was performed on an FEI XL-30 F field emission SEM, which was operated at a beam voltage of 10 kV and equipped with an EDAX energy dispersive system. Zeta potential of the nanocellulose material was measured using the same Brookhaven Instrument Corporation ZetaPALS (Holtsville, NY) utilized for DLS. A 1 g/L aqueous sample (stock solution) was tested.

### Animals

Specific pathogen-free adult male and female C57BL/6 mice (7–8 weeks) were supplied by Jackson Laboratories (Bar Harbor, ME) and weighed 20.0 ± 1.9 g when used. Animals were housed individually (one mouse per cage) receiving filtered high efficiency particulate air (HEPA) in the Association for Assessment and Accreditation of Laboratory Animal Care (AAALAC), International-accredited National Institute of Occupational and Safety Health (NIOSH) animal facility. All animals were acclimatized in the animal facility under controlled temperature and humidity environment with a 12 h light/dark cycle for one week prior to use. Beta Chips (Northeastern Products Corp., Warrensburg, NY) were used for bedding and changed weekly. Animals were supplied with water and certified chow 7913 (Harlan Teklad, Indianapolis, IN) *ad libitum*, in accordance with the guidelines and policy of the Institute of Laboratory Animal Resources, National Research Council. All procedures in the study comply with the ethical standards set forth by the Animal Welfare Act (enforced by the United States Department of Agriculture) and the Office of Laboratory Animal Welfare (OLAW). The studies were approved by the NIOSH Health Effects Laboratory Division (HELD) Institutional Animal Care and Use Committee within the Center for Disease Control (Public Health Services Assurance Number A4367-01) in accordance with an approved institutional animal protocol (protocol number 13-AS-M019).

### General experimental design

To assess pulmonary toxicity, mice were treated by repeated pharyngeal aspiration with suspensions of CNC (40 μg/mouse/day) in United States Pharmacopeia (USP) sterile water (Hospira Inc, USA). The corresponding control mice were administered USP sterile water. Mice were exposed twice a week for three consecutive weeks to obtain a cumulative dose of 240 μg/mouse of CNC. Animals were weighed and sacrificed 3 month following their last exposure. Specifically, human equivalent workplace exposure to a deposited cumulative dose of 240 μg of CNC can be achieved in ~42 working days at allowable exposure limits (5 mg/m^3^ of cellulose) defined by Occupational Safety & Health Administration (OSHA). These calculations were performed using the formula published previously by our group [24]. Briefly, the human equivalent exposure time periods required for acquiring an equivalent deposited dose as employed in this study were estimated by using alveolar lung surface areas of 102 m^2^ for human [25] and 0.06 m^2^ for mouse [26]. Other values used for these estimations included 9.6 m^3^ 8-h air intake [27]; and alveolar deposition fraction based on aerodynamic particle size. The T_1/2_ for clearance in humans is ~ 1 year and can be ignored from these estimations, as the clearance would be insignificant over the 42 days required to achieve an equivalent worker lung burden.

### Preparation and administration of CNC

Wood pulp derived CNC (freeze dried (powder form) samples) were provided by Forest Products Laboratory (FPL, United States Forest Service, Madison, WI). CNC was produced from machine-dried prehydrolysis kraft rayon-grade dissolving wood pulp by hydrolysis with 64 % sulfuric acid at 45 °C for 90 min, followed by dilution, neutralization of the acid with NaOH, and membrane filtration [28]. Commercial dissolving pulp (50 kg) as drylap is stripcut and packed into a 400 L glass-lined reactor. Under vacuum, 300 L of 64 % (PC-grade) sulfuric acid (Columbus Chemical Industries, Columbus, WI) already warmed to 45 °C is sprayed onto the cellulose over 15 min while stirring begins. After a total of 90 min at 45 °C, the reaction is quenched by dilution into 1200 L reverse osmosis (RO) water in a 6500 L glass-lined reactor. About 1 kg of (80 % technical-grade) sodium chlorite (Alfa Aesar, Ward Hill, MA) is added and stirred for a half hour. Residual chlorine dioxide is swept from the reactor headspace with a scrubber. The sulfuric acid is neutralized with 8 % sodium hydroxide solution, via the dilution of about 300 L of 50 % (FCC-grade) sodium hydroxide (Columbus Chemical Industries, Columbus, WI). The aggregated CNC suspension is circulated in a tubular ultrafiltration membrane (200,000 Dalton) system for about 40 h displacing sodium sulfate solution with RO water. The CNC, now a colloidal suspension, is then passed through a 20 micron filter before the ultrafiltration system is used to concentrate the purified CNC to 12 % solids (54 % yield) for cold storage. Some of the CNC suspension is diluted to 1 % solids into which another tenth by volume of reclaimed (85 %) tert-butanol (Aldrich, Milwaukee, WI) is mixed. This mixture is partially frozen in a commercial ice-cream machine, spread into trays then frozen solid prior to freeze drying.

Stock suspension of CNC for animal studies was prepared in USP grade water with pH adjusted to 7.0. The samples were sonicated for 2 min with a probe sonicator (Branson Sonifier 450, 10 W continuous outputs) and then sterilized by autoclaving. These stock suspensions were further diluted prior to animal exposures. Endotoxin levels in all used CNC samples were below the detection limit (0.01 EU/ml) as was assessed by a Limulus amebocyte lysate (LAL) chromogenic endpoint assay kit (Hycult biotech, Inc., Plymouth Meeting, PA).

Mouse pharyngeal aspiration was used for CNC administration. This technique has been reported to provide widespread delivery of particles to the deep lung and to be highly correlated with the administered dose [29]. Briefly, after anesthesia with a mixture of ketamine (Phoenix, St. Joseph, MO) and xylazine (Phoenix, St. Joseph, MO) (62.5 and 2.5 mg/kg subcutaneous in the abdominal area), the mouse was placed on a board in a near vertical position and the animal’s tongue was extended with lined forceps. A suspension of cellulose (40 μg/mouse) was placed posterior in the throat and the tongue held until the suspension was aspirated into the lungs. The exposure regime was repeated twice a week (Monday and Thursdays of the week @ 9 am) for three consecutive weeks. All mice from the control and CNC treated groups survived this exposure procedure and exhibited no overt behavioral or health outcomes.

### Obtaining bronchoalveolar lavage (BAL)

Mice were weighed and sacrificed 3 month post-CNC exposure with intraperitoneal injection of sodium pentobarbital (>100 mg/kg) and exsanguinated. The trachea was cannulated with a blunted 22-gauge needle, and BAL was performed using cold sterile PBS at a volume of 0.9 ml for the first lavage (kept separate) and 1.0 ml for the subsequent lavages. Approximately 5.0 ml of BAL fluid per mouse was collected in sterile 15 mL falcon tubes (Becton Dickinson Labware, Franklin Lakes, NJ). Pooled BAL cells for each individual mouse were washed in PBS by centrifugation (800 X g for 10 min at 4 °C). Cell-free, first-fraction BAL aliquots were used immediately for lactate dehydrogenase (LDH) assays, whereas the remainder was frozen at -80 °C until processed.

### BAL cell counting and differentials

The degree of inflammatory response induced by repeated CNC exposure was estimated by quantifying total cells, alveolar macrophages (AMs), polymorphonuclear leukocytes (PMNs), lymphocytes and eosinophils in the BAL. Cell counts were performed using an electronic cell counter equipped with a cell sizing attachment (Coulter model Multisizer II with a 256C channelizer, Coulter Electronics, Hialeah, FL). The cells were identified by their characteristic cell shape in cytospin preparations stained with HEMA 3 solution I, solution II and fixative (Fisher Scientific, Kalamazoo, MI), and differential counts of BAL cells was carried out. At least 300 cells per slide were considered for each sample for this examination. Additionally, quantitative assessment of giant multi-nucleated cells in BAL was performed. Blind-coded slides were independently scored by two readers. At least 2000 cells per slide were considered for each sample for this analysis.

### Preparation of lung homogenates

The whole mouse lung were separated from other tissues and weighed before being homogenized with a tissue tearer (model 985-370, Biospec Products Inc., Racine, WI) in PBS (pH 7.4) for approximately 2 min. The homogenate suspensions were aliquoted and stored at -80 °C until processed.

### Lung histopathology

Lung tissues were harvested and inflation fixed in situ with 4 % paraformaldehyde at constant pressure of 10 cm H_2_O for 10 min with the chest cavity open. Coronal sections were cut from the lungs, embedded in paraffin, and sectioned at a thickness of 5 μm with an HM 320 rotary microtome (Carl Zeiss, Thornwood, NY). Prepared sections were then stained with hematoxylin and eosin (H&E), and histological evaluation was performed. Sample identification was coded to ensure unbiased evaluation.

### Total protein activity and lactate dehydrogenase (LDH) release

Measurement of total protein in the BAL fluid and lung homogenates was performed using a modified Bradford assay according to the manufacturer’s instructions (Bio-Rad, Hercules, CA) with bovine serum albumin as the standard control. The activity of LDH was assayed spectrophotometrically using a Synergy H1 Hybrid Reader (BioTek, Winooski, VT) at 340 nm. The reduction of nicotinamide adenine dinucleotide in the presence of lactate using a Lactate Dehydrogenase Reagent Set (Pointe Scientific, Lincoln Park, MI) was monitored.

### Myeloperoxidase (MPO) activity

Inflammatory response in the lung of mice after repeated exposure to CNC was assessed by measurement of MPO in lung homogenates using commercially available enzyme-linked immunosorbent assay (ELISA) colorimetric assay (Northwest Life Science Specialties, LLC, Vancouver, WA). Results for MPO activity were normalized to total protein content in tissue homogenate samples.

### Evaluation of oxidative stress biomarkers

Oxidative damage to the lung following repeated CNC exposure was evaluated by the presence of lipid peroxidation products, protein carbonyls, vitamin E and total antioxidant reserve in tissue homogenates. HNE-His adducts, lipid peroxidation end products, were measured in lung homogenates by ELISA using the OxiSelect hydroxynonenal (HNE-His) adduct kit (Cell Biolabs Inc., San Diego, CA). The quantity of HNE-His adducts in protein samples were evaluated by comparing its absorbance with that of a known hydroxynonenal-bovine serum albumin (HNE-BSA) standard curve. The quantity of oxidatively modified proteins in lung homogenates, as assessed by measurement of protein carbonyls, was determined using the Biocell PC ELISA kit (Northwest Life Science Specialties). The amount of protein carbonyls was measured spectrophotometrically at 450 nm. The level of vitamin E in lung homogenates was determined using VE Elisa kit (Biotang Inc., Lexington, MA) according to manufacturer’s instructions. Extracts of vitamin E from lung homogenates were prepared using a procedure described by Lang and his co-workers [30], prior to the measurements. Total antioxidant capacity in lung homogenates was measured using NWLSS TAC-Peroxyl assay kit (Northwest, Vancouver, WA) according to the manufacturer’s instructions. The degree of chemiluminescence quenching is proportional to the radical trapping ability of the antioxidant sample. The luminescence was recorded using a luminometer (Synery H1, Winooski, VT) at 1 s integration time for a 15 min period until the last maximal plateau was reached. The antioxidant concentration of samples was determined by comparing induction time to that of a known standard curve.

### Measurement of cytokines

Cytokines in the BAL fluids from mice exposed to repeated CNC exposure were analyzed using a Bio-Plex system: 23-Plex cytokines/chemokines and TGF-β 3-plex assays (Bio Rad, Hercules, CA). The concentrations were calculated using Bio-Plex Manager 6.1 software (Bio-Rad, Hercules, CA) based on standard curves.

### Lung collagen measurements

Total lung collagen content was determined by quantifying total soluble collagen using the Sircol Collagen Assay kit (Accurate Chemical and Scientific Corporation, Westbury, NY). Briefly, lungs were homogenized in 0.7 ml of 0.5 M acetic acid containing pepsin (Accurate Chemical and Scientific Corporation, Westbury, NY) with 1:10 ratio of pepsin: tissue wet weight. Each sample was stirred vigorously for 24 h at 4 °C, centrifuged, and 200 μl of supernatant was assayed according to the manufacturer’s instructions.

### Estrogen (E) level evaluation

Level of the endogenic mouse estrogen (E) concentration in the serum of mice exposed to CNC was assessed using E Elisa kit (My BioSource, San Diego, CA) according to the manufacturer’s instructions. This assay employs the competitive inhibition enzyme immunoassay technique. The estrogen concentration of samples was determined from a standard curve.

### Airway hyperresponsiveness evaluation

The responsiveness of mouse airways was measured 3 month post-CNCexposure using a non-invasive whole-body plethysmograph (WBP) system (Buxco Systems Inc., Troy, NY). Briefly, mice were placed in WBP of approximately 300 mL in which the animals were unrestrained and exposed to increasing concentrations (10, 25, 50 mg/ml) of aerosolized methacholine (MCh; Sigma Aldrich, St. Louis, MO). After the baseline measurements, MCh is nebulized for 1.5 min, and pressure changes within the chamber due to respiration were monitored and recorded (2 min/concentration). The pressure signals were post-processed using Matlab (Mathworks, Inc.) to calculate airway reactivity and expressed as enhanced pause (Penh) values. Penh is a reasonable analogue of airway responsiveness to a non-specific inhaled stimulus, such as MCh, which provides an accepted measure for comparison between the experimental groups.

### Total RNA extraction, microarray hybridization & statistical analysis of microarray data

Global gene expression for each treatment was determined using high-throughput mRNA microarray analysis following Minimum Information About a Microarray Experiment (MIAME) guidelines. Total RNA was isolated from left lung tissues of control and CNC exposed male and female mice 3 months post exposure (*n* = 5 for each group). The RNA was isolated using TRIzol reagent (Invitrogen, Carlsbad, CA, USA) and purified using RNeasy MiniKits (Qiagen, Mississauga, ON, Canada) as described by the manufacturer. On-column DNase treatment was applied (Qiagen, Mississauga, ON, Canada). All RNA samples showing A260/280 ratios between 2.0 and 2.15 were further analyzed for RNA integrity using an Agilent 2100 Bioanalyzer (Agilent Technologies, Mississauga, ON, Canada). RNA integrity numbers above 5.0 were used in the experiment. Total RNA was stored at − 80 °C until microarray analysis. A total of 200 ng of RNA from each sample (5 per treatment group) was analyzed by microarray hybridization on Agilent 8 × 60 K oligonucleotide microarrays (Agilent Technologies Inc., Mississauga, ON, Canada). Data were acquired using Agilent Feature Extraction software version 9.5.3.1. A reference design [31–33] with the median signal intensities was used for the microarray data analysis. Five biological replicates per experimental condition and for each gender were considered for the analysis. The background for each array was measured using the (-)3xSLv1 probe. Spots with median signal intensities less than the trimmed mean (trim = 5 %) plus three trimmed standard deviations of the (-)3xSLv1 probe were flagged. All pre-processing of the data was conducted using R [34]. The data was normalized using the global lowess method [35] using the transform.madata function in the MAANOVA library [36]. The background estimates as well as boxplots, cluster analysis and principle component analysis were used to identify microarrays with poor data quality and outliers. Differentially expressed genes were identified using the Fs statistic [37] in the MAANOVA library for each time point independently. The *p*-values were estimated using the permutation method (30,000 permutations with residual shuffling). The least-squares means [38, 39], a function of the model parameters was used to estimate the fold changes for each pairwise comparison. Genes showing expression changes of at least 1.2-fold in either direction compared to their matched controls and having p-values of less than or equal to 0.05 (p ≤ 0.05) were considered significantly differentially expressed and were considered for further analysis. As most of the cytokine protein levels in the lungs were similar in trend to those observed in their corresponding mRNA levels in microarrays as well as consistent with their differences between males versus females (e.g., Eotaxin or CCL11, MIP-1β, IL-6, IL-12p70, Collagens), validation of microarray data by RT-qPCR was not considered necessary as part of the current study.

### Gene Ontology (GO), pathway analysis and prediction of transcriptional factors

The gene ontology (GO) and Kyoto Encyclopedia of Genes and Genomes (KEGG) pathway enrichments associated with differentially expressed genes was performed using Database for Annotation, Visualization and Integrated Discovery (DAVID) v6.7 software [40, 41]. To obtain an overview of perturbed functions in the lungs upon exposure to CNC in male and female mice, the Cellular Component, Biological Process and Molecular Function annotations were classified into broad groups based on the GO-slim classification system using CateGOrizer [42]. Further the CateGOrizer outputs were directly exported to Reduce Visualize Gene Ontology (REViGO) for a semantic representative subset analysis of non-redundant GO terms [43]. The prediction of the key transcriptional factors which regulated the expression of the upregulated differentially expressed genes (DEG)s in males and females upon exposure to CNC was performed using ChEA (ChIP Enrichment Analysis) software tool [44]. A *P* value cutoff of ≤ 0.05 was considered significant in each case.

### Statistical analysis

Results were compared by One Way ANOVA using the all pairwise multiple comparison procedures (Holm-Sidak method). All results are presented as mean + SEM. *P* values of less than 0.05 were considered to indicate statistical significance.

## Results

### CNC characterization

**Fig. 1:**
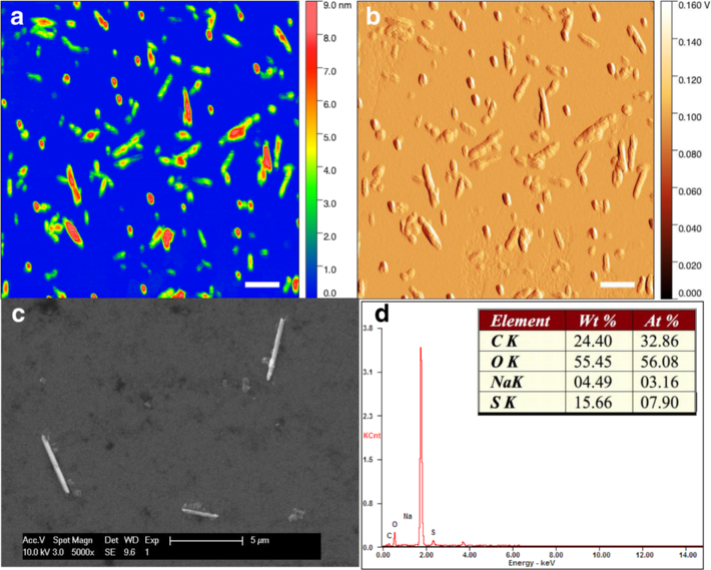
Imaging of CNC using AFM height (**a**) and amplitude (**b**) modes, and SEM (**c**). The EDX spectrum is also shown (**d**), along with the calculated weight percentages (Wt%) and atomic percentages (At%). Silicon elemental contributions are ignored in the EDX analysis. Scale bars are 200 nm for **a** and **b**

AFM imaging revealed the presence of nanoscale particles with an average length of 158 ± 97 nm and average width of 54 ± 17 nm, based on 200 particle measurements (Fig. 1a-b). DLS determined the hydrodynamic diameter to be 149.8 ± 2.6 nm, in good agreement with AFM findings. A similar fibril morphology was observed using SEM, albeit on a larger scale, possibly due to aggregation during the SEM sample preparation (Fig. 1c). The elemental analysis performed using EDX produced expected atomic percentages, with oxygen as the most abundant element, followed by carbon, sulfur, and sodium (Fig. 1d). The presence of sulfur can be attributed to the production process for the CNC, whereby organosulfate groups are formed during the cellulose hydrolysis using sulfuric acid, and sodium-containing solutions may be utilized during the neutralization step. For clarity, elemental analysis ignored any observed silicon, as its presence was attributed to the use of a silicon wafer. Zeta potential of the nanocellulose material was determined to be -68.26 +/- 2.89 mV. The negative surface charge of the nanocellulose is expected due to the presence of deprotonated organosulfate functionalities introduced during the manufacturing process.

### CNC induced pulmonary damage, inflammatory cell recruitment and cytokine responses

**Fig. 2:**
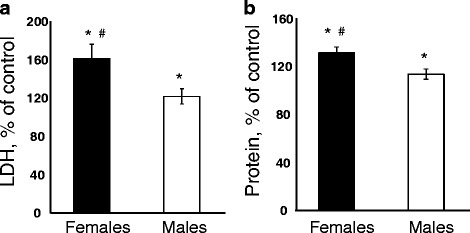
Pulmonary tissue damage measured as LDH (**a**) and Protein (**b**) in BAL of C57BL/6 mice 3 months after repeated exposure to CNC (cumulative dose of 240 μg/mouse). Black columns – female mice exposed to CNC, open columns – male mice exposed to CNC. Mean absolute values of LDH in BAL from PBS-exposed control male and female mice were 53.8 ± 2.9 U/L and 52.3 ± 2.1 U/L, respectively. Mean absolute values of protein level in BAL from PBS-exposed control male and female mice were 0.238 ± 0.005 mg/ml and 0.224 ± 0.009 mg/ml, respectively. No significant differences were found between levels of LDH and protein measured in the BAL of controls from male and female mice. Mean ± SEM (*n* = 10 mice/group). *p < 0.05, vs control PBS-exposed mice, ^#^ *p* < 0.05, vs male mice exposed to CNC

Assessment of pulmonary damage following 3 months post CNC exposures administered by pharyngeal aspiration revealed that exposure to CNC caused substantial lung damage in both male and female mice with higher damage observed in female mice. In particular, level of LDH (67 % vs 21 %) and total protein content (31 % vs 13 %) were increased significantly in female compared to male treated ones, respectively (*p* < 0.05; Fig. 2a, b).

**Fig. 3:**
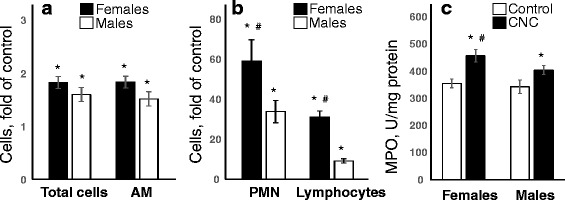
Pulmonary inflammation evaluated by assessment of (**a**, **b**) cell profile from BAL of mice and (**c**) activity of myeloperoxidase (MPO) in lung homogenates of C57BL/6 mice 3 months after repeated pharyngeal aspiration with CNC (cumulative dose of 240 μg/mouse). **a**, **b** Black columns – female mice exposed to CNC, open columns – male mice exposed to CNC. Mean absolute values from PBS-exposed control female mice were (394.4 ± 28.6)x10^3^, (373.8 ± 26.5)x10^3^, 430.0 ± 214.0, and 396.0 ± 199.0 for total cells, alveolar macrophages, PMNs and lymphocytes, respectively. Mean absolute values from PBS-exposed control male mice were (343.6 ± 40.7)x10^3^, (341.3 ± 40.2)x10^3^, 645.3 ± 204.0, and 899.4 ± 218.0 for total cells, alveolar macrophages, PMNs and lymphocytes, respectively. No significant differences were found between number of total cells, alveolar macrophages, PMNs and lymphocytes in controls from female and male mice. Mean ± SEM (*n* = 10 mice/group). *p < 0.05, vs control PBS-exposed mice, ^#^ *p* < 0.05, vs male mice exposed to CNC. **c** Open columns – control mice, black columns –mice exposed to CNC. No significant differences were found between levels of MPO measured in the lung homogenates from females and males control mice. Mean ± SEM (*n* = 10 mice/group). *p < 0.05, vs control PBS-exposed mice, ^#^ *p* < 0.05, vs male mice exposed to CNC

**Fig. 4:**
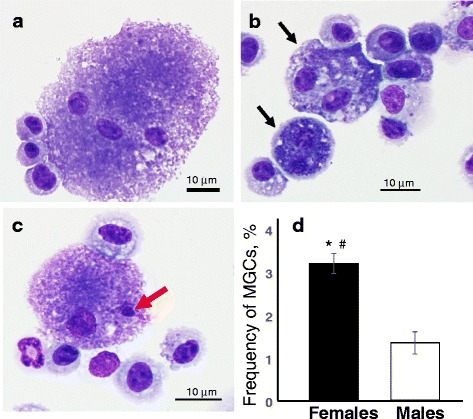
Representative light micrographs of giant multi-nucleated (**a**-**c**) cells including bi-nucleated (**b**) and micro-nucleated (**c**) alveolar microphages from BAL fluids of female mice 3 month post repeated exposure with CNC (marked by arrows). Frequency of giant multi-nucleated cells (MGCs) in BAL fluids of female or male mice 3 month after the last exposure with CNC (**d**). Black columns – giant BAL cells from female mice exposed to CNC, open columns– giant BAL cells from male mice exposed to CNC. Mean ± SEM (*n* = 10 mice/group). *p < 0.05, vs control PBS-exposed mice, ^#^ *p* < 0.05, vs male mice exposed to CNC. Blind-coded slides were independently scored by two readers. A total of 2000 cells per sample were scored. No MGCs were found in control mice

**Fig. 5:**
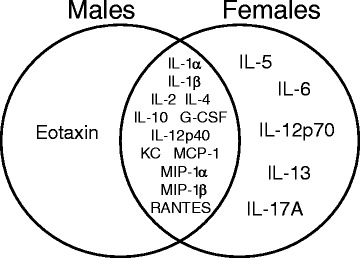
A Venn diagram presenting differential cytokines/chemokines responses 3 month post repeated exposure of female and male C57BL/6 mice to CNC (cumulative dose of 240 mg/mouse). These measurements were performed using Bio-Rad 23-plex mouse assay kit, composed of a combination of pro- and anti- inflammatory cytokines with a subset of chemokine’s

Exposure to respirable CNC caused significant rise in total cell numbers and macrophages in BAL irrespective of the gender (Fig. 3a). However, CNC-exposed female mice showed a significantly higher fold increase in total PMN and lymphocytes compared to male mice (59 vs 34) and (31 vs 9), respectively (Fig. 3b). The increase in the activity of MPO, an abundant leukocyte protein that generates reactive oxidants, was greater in the female lungs compared to that observed in male mice (Fig. 3c). Multi-nucleated giant cells (MGCs), merged monocytes and/or macrophages [45] reflective of chronic inflammation were found in BAL fluid of mice after 3 months following CNC exposure (Fig.4a-c). The number of MGCs found in BAL cells recovered from females was 2.8 fold higher compared to that observed in males (Fig. 4d). Several pro-inflammatory cytokines were increased following exposure to CNC, likely contributing to the development and/or progression of CNC-induced lung inflammation. Panel of inflammatory, adhesive molecules and immunogenic mediators are listed in Table 1. Following 3 months post exposure to CNC, the fold-increase of the levels of IL-1β, IL-10, IL-12p70, KC, MCP-1, MIP-1α and MIP-1β were significantly greater in BAL fluids of females compared to male mice. Other cytokines including IL-1α, IL-2, IL-4, IL-2p40, G-CSF and RANTES were increased in BAL fluids of both genders (Table 1). While the expression of Eotaxin was only found in CNC exposed male mice, the increase in the levels of cytokines IL-5, IL-6, Il-12p70, IL-13 and IL-17A were unique to CNC exposed female mice (Fig. 5).

**Table 1:** Comparison of the levels of inflammatory cytokines and chemokines in the BAL fluid of C57BL/6 female and male mice 3 months after repeated exposure with CNC

Cytokines	Control (pg/ml)	Females (fold of control)	Males (fold of control)
IL-1α	1.11 ± 0.05	1.78 ± 0.08^*^	1.87 ± 0.10^*^
IL-1β	24.75 ± 0.70	2.21 ± 0.12^*#^	1.68 ± 0.08^*^
IL-2	1.95 ± 0.11	1.73 ± 0.12^*^	1.69 ± 0.19^*^
IL-4	2.68 ± 0.03	1.34 ± 0.04^*^	1.29 ± 0.02^*^
IL-5	1.77 ± 0.10	1.87 ± 0.11^*#^	0.85 ± 0.09
IL-6	3.92 ± 0.17	1.29 ± 0.08^*^	1.08 ± 0.05
IL-10	6.83 ± 0.27	1.53 ± 0.08^*#^	1.28 ± 0.12^*^
IL-12(p40)	110.30 ± 7.11^α^	3.80 ± 0.26^*^	3.20 ± 0.27^*^
	66.76 ± 1.99^β^		
IL-12(p70)	24.95 ± 0.80	1.36 ± 0.07^*#^	1.17 ± 0.04
IL-13	113.39 ± 3.09	1.27 ± 0.04^*^	1.16 ± 0.05
IL-17A	4.20 ± 0.20	1.51 ± 0.06^*#^	0.99 ± 0.08
Eotaxin	468.96 ± 32.56	1.15 ± 0.07	1.32 ± 0.07^*^
G-CSF	6.77 ± 0.19	1.32 ± 0.05^*^	1.18 ± 0.08^*^
KC	7.73 ± 0.21	4.16 ± 0.56^*#^	2.33 ± 0.18^*^
MCP-1	29.30 ± 0.72	8.15 ± 1.7^*#^	2.35 ± 0.26^*^
MIP-1α	5.81 ± 0.16	13.43 ± 1.63^*#^	6.51 ± 0.64^*^
MIP-1β	3.50 ± 0.18	2.17 ± 0.12^*#^	1.42 ± 0.09^*^
RANTES	2.37 ± 0.22	1.87 ± 0.15^*^	1.90 ± 0.28^*^

The control values otherwise noted correspond to the mean absolute values (pg/ml) from PBS-exposed male mice. The PBS-exposed control female and male mice were not significantly different, except for IL-12(p40). Level of IL-12(p40) for control females^α^ mice (110.30 ± 7.11 pg/ml) was significantly different as compared to control males^β^ mice (66.76 ± 1.99 pg/ml). For comparison, cytokines data presented as folds of control for female and male mice exposed to CNC. Mean ± SEM (*n* = 10 mice/group). *p < 0.05, vs control PBS-exposed mice, ^#^ *p* < 0.05, vs male mice exposed to CNC

### CNC induced oxidative stress

**Fig. 6:**
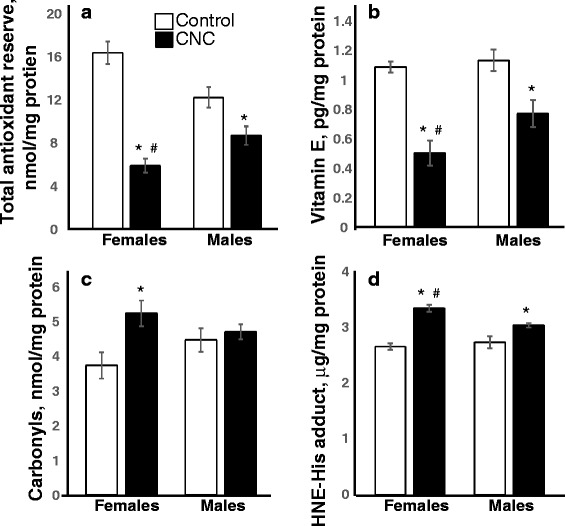
Oxidative stress evaluated by measurements of **a** total antioxidant reserve, **b** vitamin E, **c** protein carbonyls and **d** HNE-His adduct in the lung homogenates of C57BL/6 mice 3 months after repeated exposure to CNC. Open columns – control mice, black columns – mice exposed to CNC. Mean ± SEM (*n* = 5-10 mice/group). *p < 0.05, vs control PBS-exposed mice, ^#^ *p* < 0.05, vs male mice exposed to CNC

Several markers of oxidative stress including total antioxidant reserve, level of carbonyls, fat soluble antioxidant, vitamin E, and lipid peroxidation products, 4-hydroxynonenal (HNE), were measured in the lungs of female and male mice following 3 months post repeated CNC exposure (Fig. 6). Total antioxidant reserve was 2.8-fold lower after CNC exposure in female mice compared to 1.4-fold lower levels seen in males (Fig. 6a). Level of vitamin E in female mice was decreased 2.2-fold compared to 1.4-fold in males (Fig. 6b). CNC caused a 1.4-fold accumulation of protein carbonyls in female mice, while no significant changes were found in the lungs of male mice (Fig. 6c). 4-HNE, a common byproduct of lipid peroxidation was increased in females and males 1.2 and 1.1 fold, respectively (*p* < 0.05; Fig. 6d).

### CNC facilitated increase of TGF-β1 and collagen accumulation

**Fig. 7:**
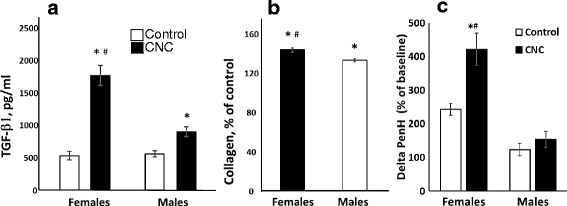
Levels of TGF-β1 in the BAL (**a**), levels of collagen (**b**) and airway reactivity to direct stimulation of MCh (**c**) measured in the lung of female and male C57BL/6 mice 3 month post repeated exposure to CNC (cumulative dose of 240 μg/mouse). **a** & **c** Open columns – control mice, black columns – mice exposed to CNC. Mean ± SEM (n ≥ 5 mice/group). *p < 0.05, vs control PBS-exposed mice, ^#^ *p* < 0.05, vs male mice exposed to CNC. **b** Black columns – female mice exposed to CNC, open columns – male mice exposed to CNC. Mean absolute values of collagen in the lung of PBS-exposed control male and female mice were not significantly different (36.02 ± 1.90 μg/mg lung and 32.20 ± 1.50 μg/mg lung, respectively). Mean ± SEM (*n* = 10 mice/group). *p < 0.05, vs control PBS-exposed mice, ^#^ *p* < 0.05, vs male mice exposed to CNC

TGF-β1 is a pleiotropic cytokine secreted by a number of immune cells including macrophages. We found that elevation of TGF-β1 was significantly greater in BAL fluids of females compared to that found in male mice. In particular, level of TGF-β1 in female mice was 3.2 fold higher compared to that seen in males (Fig. 7a). Collagen increase in mouse lungs was also significantly greater in females compared to males (Fig. 7b).

### Airway responsiveness to direct stimulation of MCh

Airway reactivity in responses to direct stimulation of MCh challenge is depicted in Fig. 7c. Data are shown as percent delta Penh, representing the difference between the maximum value registered post-MCh challenge at 50 mg/ml concentration and the baseline Penh in each case. Although modest increases in Penh after MCh challenge were also observed in controls, the magnitude of the response was significantly altered in CNC exposed mice. Most importantly exposure to CNC significantly (*p* < 0.05) increased airway responsiveness to MCh as compared to control (PBS) mice by ~1.7-fold only in females (Fig. 7c). No significant change in airway reactivity was observed at 50 mg/ml concentration of MCh in male mice compared to their respective controls. Interestingly the overall airway reactivity responses to direct stimulation of MCh challenge were elevated in both female control mice as well as CNC exposed mice, compared to males (Fig. 7c).

### Histopathology

**Fig. 8:**
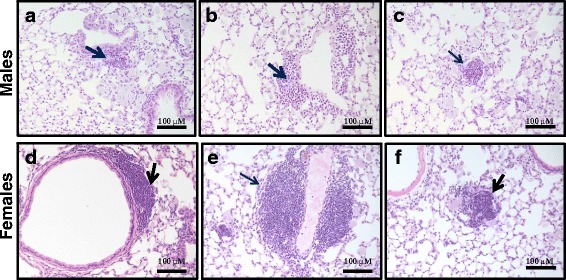
Light micrographs of H&E stained sections from lungs of female and male C57BL/6 mice 3 month post CNC repeated exposure showing chronic pulmonary inflammation: peribronchial (**a** & **d**), perivascular (**b** & **e**) and parenchymal (**c** & **f**)

**Fig. 9:**
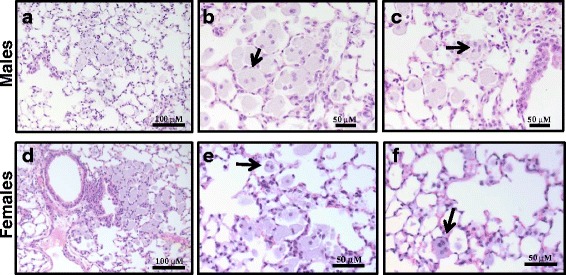
Light micrographs of H&E stained sections from lungs of female and male C57BL/6 mice 3 month post CNC repeated exposure showing giant alveolar macrophages (**a** & **d**), bi- (**b** & **e**) and multi- nucleated (**c** & **f**) cells

Microscopic sections of the lungs in the control animal groups (both males and females) reveal normal histology of conductive and respiratory airways. Three months post repeated CNC exposure microscopic sections of male and female lungs reveal chronic peribronchial and perivascular inflammation (Fig. 8), and numerous alveolar macrophages, including multi-nucleated forms (Fig. 9). The degree of perivascular inflammation was higher in females than in males.

### Differentially expressed genes in males versus females upon exposure to CNC

**Fig. 10:**
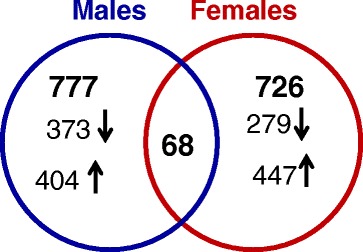
Overlap of differentially expressed genes from both genders of C57BL/6 mice 3 months after repeated exposure to CNC

Global pulmonary gene expression changes were assessed in the lung tissue of males and females 3 months post exposure to CNC. A total of 845 and 794 of the 22,486 probes were significantly differentially expressed (≥1.2-folds, p ≤ 0.05) in male and female mice, respectively (Additional file 1: File S1). Figure 10 shows the overlap of DEGs across both genders. A total of 68 genes were found to be commonly regulated in the lungs upon exposure to CNC in males (~8.0 %) and females (~8.5 %). A small overlap in the DEGs between males and females suggests that pulmonary outcomes and toxicity associated with CNC exposures could be gender dependent.

### Analysis of GO terms associated with DEGs of CNC exposure in males versus female mice

**Fig. 11:**
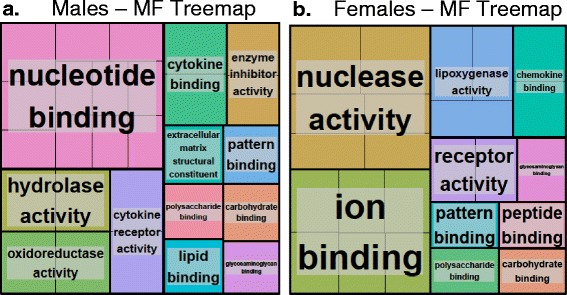
The enrichment of differentially expressed genes among molecular function category in mice exposed to CNC. Treemaps of DEGs in **a** males and **b** females generated using REVIGO. Each rectangle is a single cluster representative. The representatives are joined into ‘superclusters’ of loosely related terms, visualized with different colors. Size of the rectangles reflects either the p-value, or the frequency of the GO term in a given cluster. For more details please refer to methods section

A detailed GO enrichment analysis of every biological process (BP), molecular function (MF) and cellular component (CC) associated with DEGs upon exposure to CNC in male and female mice was conducted using DAVID [40, 41]. A significant number of GO terms in each category were found to be commonly enriched upon CNC exposure in both males and females (Additional file 2: Table S1 and Additional file 3: File S2). The enriched GO-terms commonly found in both males and females (Additional file 2: Table S1) generally included housekeeping genes, including those for cellular organization and biogenesis (GO:0016043), metabolism (GO:0008152) and development (GO:0007275) processes, response to stress/stimuli (GO:0006950, GO:0009605), cell communication (GO:0007154) and signal transduction (GO:0007165). The analysis of non-redundant GO-terms using ReVIGO [43], indicated the enrichment of MFs related to nuclease activity, ion- and chemokine binding, lipoxygenase- and receptor activity in females; and enrichment of nucleotide/cytokine binding, hydrolase-/oxidoreductase-/cytokine receptor-/enzyme inhibitor activity functions in males (Fig. 11). In particular, enriched categories under MF and CC together implicated the abundance of intracellular/cytoplasmic genes and their crucial role in biological processes focused on cellular development/function/growth and response to stimuli in males (Additional file 2: Table S1 and Fig. 11A). Similarly in females, the abundance of genes localized in extracellular and plasma membrane regions reflects their overrepresentation in carrying out biological functions related to cell adhesion, cellular metabolism/catabolism and inflammation (Additional file 2: Table S1 and Fig. 11B). Interestingly, the involvement of many DEGs in carbohydrate/pattern/polysaccharide and glycosaminoglycan binding were found to be commonly enriched between both males and females. This suggests that irrespective of gender (Fig. 11), one can specifically detect changes in gene expression profiles that are reminiscent of effects in response to other small molecules similar to cellulose nanomaterials such as chitin, amino glycan’s, poly saccharides etc.

### KEGG pathway enrichment analysis

The KEGG pathway enrichment analysis was performed for both up- and downregulated DEGs in males and females 3 months post exposure to CNC particles. While both up- and down-regulated DEGs in males were commonly found to be mostly enriched in circadian rhythm signaling, the KEGG pathways involved in metabolism (e.g, fructose and mannose, drug, retinol) and calcium signaling were only significantly enriched by upregulated DEGs; and pathways related to cancer (e.g., bladder, prostate) and inflammatory response (e.g., cytokine-cytokine receptor interaction, chemokine signaling, phagocytosis, leukocyte transendothelial migration, antigen presentation and processing) were significantly enriched by downregulated DEGs (Additional file 4: Table S2). In females, upregulated genes were mostly enriched in inflammatory and immune response signaling (e.g., chemokine signaling, cytokine-cytokine receptor interactions, endocytosis), while downregulated genes were significantly enriched in gap junction, melanogenesis, axon guidance, calcium and chemokine signaling pathway (Additional file 4: Table S2). Further analysis of the enriched KEGG pathways suggested specific differences in the responses of males and females. While the cytokine-cytokine receptor interaction and chemokine signaling pathways were significantly enriched by upregulated genes in females, in males they were significantly enriched by downregulated DEGs. These results clearly highlight gender specific differential regulation of pathways and signaling mechanisms related to inflammatory responses upon exposure to CNC materials.

### Significant transcriptional factors for upregulated DEGs

Considering that most changes in gene expression are controlled by upstream regulatory transcriptional factors, we searched on ChEA for transcriptional factors that could regulate the upregulated genes identified both in males and females. A total of 70 transcriptional factors in males and 74 in females were predicted to significantly (*p* < 0.005) modulate upregulated DEGs in each case (Additional file 5: File S3). Most importantly, the regulation of transcription factors related to oxidative stress mechanism and antioxidant responses (e.g., NRF2, ESR1, SOX2, NR1I2, KLF2/4/5) were most significant in males, the regulation of factors related to cell cycle/death and differentiation (e.g., KDM5B, TAL1, TRIM28, CREM, SMARCA4) as well as immune/inflammatory responses (e.g., CEBPB, CREB1) were significant in females. Disregarding the overlapping transcriptional factors between males and females, the regulators of cell cycle/death/differentiation including hematopoiesis (e.g., ZFP42, SFPI1, GATA1, FLI1, CCND1, RUNX1, LMO2) in females; and oxidative-stress mediated antioxidant responses (e.g., ESR1, NR1I2, TET1, BMI1) responses in males, were overrepresented by the upregulated DEGs (Additional file 5: File S3). Overall the transcription regulator analysis highlights significant enrichment of factors related to antioxidant mechanisms triggered in response to oxidative stress in males upon exposure to CNC, and not females.

### Inflammatory and oxidative stress responses

Inflammatory and antioxidant responses were among the most perturbed processes in the lungs upon exposure to CNC in females and males, respectively. Specifically, the inflammatory responses in females were in part driven by several cytokines/chemokines, including CXCL1, CCL3 and CCL4; and inflammatory genes such as PKCB, TNFSR4, CXCR6, LIF, IL2RB, ITK and ARRB. A significant upregulation of genes corresponding to negative acute phase response (APR) including APOA2, AHSG and ALB was observed in the lungs of male mice (Additional file 1: File S1). However, no differential regulation of these negative APR genes were found in females, further supporting the enrichment of immune/inflammatory BP and MF GO categories in females than males. Decreased inflammation in males - compared to females - is also corroborated by the upregulation of several antioxidant enzymes. A significant upregulation of genes related to phase I and II antioxidant/detoxification enzymes including CYP2E1, GSTA5, NQO1, EPHX (XDH) as well as antioxidant protein superoxide dismutase (SOD) was found only in the lungs of male mice. Thus, a trend for lack of antioxidant enzymes and/or proteins in females may contribute to the greater oxidative damage that could be a cause or consequence of inflammation.

## Discussion

The high scale production of CNC and their growing applications in composite materials, electronics, food containers and appliances has gained increasing attention due to their high strength and stiffness combined with low weight, biodegradability and sustainability. While CNC hold great commercial potential, data regarding their health effects and safety are missing. The lungs are the primary route for particulates entrance into the human body. Several studies have reported pathological manifestation showing epithelial hyperplasia, granulomatous lesions, and fibrosis in rodent lungs following exposure to cellulose fibers [7, 9, 46–48]. Recent evidence also suggests that occupational exposure to CNC, nanosized cellulose particles, could prime adverse pulmonary effects. The airborne CNC could be released during production and/or handling at the workplace [49, 50]. Bio-persistence of cellulose fibers shown in rat lungs could aggravate respiratory outcomes causing airway ailments [51–54]. The morphology, high aspect ratio and small aerodynamic diameter of CNC makes it possible to be effectively deposited in the gas exchange alveolar area. A recently published study demonstrated that CNC is highly biodurable and could be biopersistent [55]. Previously, we found that bolus administration of respirable CNC to mice elicited dose-dependent oxidative stress, tissue damage, and robust inflammatory responses in the lungs. In this study, we investigate the pulmonary outcomes induced by repeated CNC cumulative exposure to 240 μg/mouse. We found that CNC caused impaired lung functions, pulmonary inflammation and damage. Accelerated oxidative stress, elevated TGF-β, and collagen deposition were observed in the lungs of CNC exposed mice. Interestingly, all these changes were highly expressed in female compared to male mice, clearly highlighting gender based differences in pulmonary responses upon exposure to CNC. To the best of our knowledge, the current study is the first to report or point out these pulmonary differences in male and female mice upon exposure to CNC materials.

The current Occupational Safety and Health Administration (OSHA) permissible exposure limit (PEL) set for cellulose is 15 mg/m^3^ (total dust) and 5 mg/m^3^ (respirable fraction) as 8 h time weighted average (TWA) concentration (29 CFR [56] 1910.1000, Table Z-1). Thus, under manufacturing settings, lung burdens comparable to those used in this study (adjusted by lung surface area) can be achieved by workers in 42 working days (5 mg/m^3^, lung ventilation of 9.6 m^3^/day [27] and deposited pulmonary fraction of 20 % [57]). Moreover, the doses of CNC utilized in the current study (240 μg/mouse) are relevant to the actual workplace and are certainly less than those that could be achieved during lifetime work exposures (8 h/d, 5 d/wk, 45 yr, 29 CFR [56]) [58].

Previous studies have reported gender based differences in the expression of pro-inflammatory cytokines [59–62]. Cellular responses to airborne particulates are orchestrated by release/production of a number of inflammatory mediators. We found that the majority of cytokines/chemokines including IL-1α, IL-1β, IL-2, IL-4, KC, MCP-1, MIP-1α, MIP-1β, G-CSF, IL-10, IL-12p40, and RANTES were up-regulated in both genders upon exposure to CNC (Table 1). Increased release of cytokines/chemokines is also consistent with the recruitment of phagocytes, e.g., monocytes/macrophages neutrophils, and lymphocytes (Fig. 5). While Eotaxin was found to be uniquely expressed in the lungs of CNC exposed male mice, the up-regulation of IL-5, IL-6, IL-13, IL-12p70, and IL-17A was observed only in females (Table 1, Fig. 5). Notably, IL-17 family cytokines, IL-17A and IL-17 F, that target innate immune cells and epithelial cells to produce G-CSF and IL-8 (CXCL8), are known to induce increased neutrophil production and recruitment. Recently, IL-17 has been shown to be associated with the development of lung inflammatory diseases such as chronic obstructive pulmonary disease (COPD). IL-17A is essential to the development of elastase-induced neutrophilic inflammation and lung emphysema, which was associated with increased levels of neutrophil-related chemokines such as KC, MIP-2 and IL-1β [63]. Recently, it has also been reported that overexpression of IL-17A induces mucus metaplasia via IL-13 [64]. Moreover, repeated exposure to CNC caused development of chronic peribronchial and perivascular pulmonary inflammation with deposition of numerous bi-nucleated and multinucleated alveolar macrophages in the lungs of both genders (Figs. 8 and 9). However, the degree of perivascular inflammation was higher in females. Compared to males, female mice clearly indicated an increased inflammatory response upon exposure to CNC materials. This is further supported by the GO enrichment, KEGG pathway (Additional file 4: Table S2) and upstream transcription factor regulator analysis based on significantly overexpressed genes in females (Additional file 5: File S3). In females, the enrichment of GO MF categories (e.g., cytokine binding, cytokine-receptor activity, inflammation), KEGG pathways (e.g., chemokine signaling, cytokine-cytokine receptor interactions, endocytosis) and/or the prediction of regulatory factors (e.g., CEBPB, CREB1) involved in inflammatory and immune responses was apparent. Because oxidative stress can be the cause or consequence of inflammation, the contribution of gender difference in pro-inflammatory cytokines to gender difference in oxidative damage upon CNC exposure warrants further investigation.

CNC exposure also caused chronic pulmonary inflammation with manifested accumulation of MGCs in the lungs (Figs. 4 and 9). MGCs have been regarded as hallmark of chronic inflammation. Accumulation of MGCs have reported to play a role in hard metal lung disease leading to interstitial pneumonia and centrilobular fibrosis [65]. MGCs are also important mediators of tissue remodeling and repair and are also responsible for removal or sequestration of foreign material, intracellular bacteria and non-phagocytosable pathogens [66]. It has been reported previously that macrophage fusion factors (e.g., IL-4, IL-13 and α-tocopherol) and identified adhesion receptors and signaling intermediates, as well as an adhesion protein substrate (vitronectin) supported macrophage fusion forming MGC [45]. Studies on the molecular mechanism of macrophage fusion have revealed it to be a mannose receptor mediated phagocytic process [45, 67, 68]. In particular, IL-4 and IL-13, the two potent macrophage fusion factors, have been reported to up-regulate the expression of the mannose receptor that is essential for macrophage fusion [65, 66]. Moreover, the effects of IL-13 and IL-4 were neither additive nor synergistic, and the maximum fusion was observed when both IL-4 and IL-13 were present. This is in line with the current study where overexpression of both IL-4 and IL-13 in females lead to increased MGCs compared to males that only had overexpression of IL-4 (Fig. 5). Further, the GO enrichment of “pattern/polysaccharide/glycosaminoglycan binding” MFs identified by DEGs in the lungs of both males and females (Fig. 11) also highlights and corroborates the known function role of mannose receptors as a pattern recognition receptors [69, 70]. These endocytic/phagocytic receptors are known to recognize patterns of carbohydrates/structural units on the surfaces and cell walls of microbial and infectious agents. Based on this, we suggest that the recognition and uptake mechanisms of CNC materials could be similar to molecules having repeating structural units such as chitin, polysaccharides or peptidoglycans commonly found in microbial cell walls and viral particles - virions. Exposure to such materials has been previously shown to induce robust inflammatory and oxidative stress responses leading to infection, asthma, COPD and other lung maladies.

**Fig. 12:**
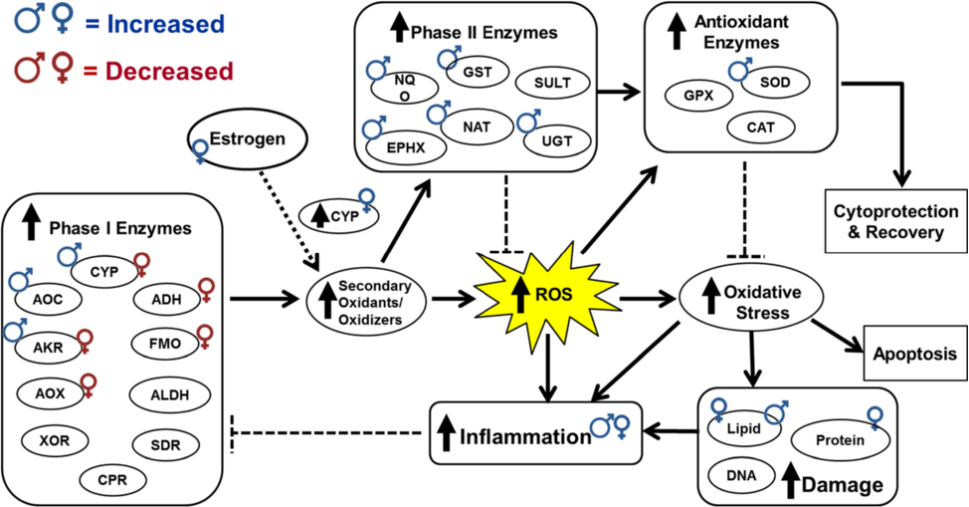
Schematic representation of the detoxification/antioxidant mechanisms and biological effects associated with oxidative damage upon pulmonary exposure to CNC in male and female mice

As the primary target organ to airborne/inhaled materials, lungs play a major role in metabolizing such substances with the aim of reducing their potential toxicity. The metabolism of naturally occurring and/or xenobiotic organic compounds is often divided into two groups, called phase I and phase II. In this study, exposure to CNC materials was shown to alter the expression of both phase I and II enzymes in the lungs, albeit with differences between males and females. In phase I reactions, a variety of enzymes introduce reactive and polar groups (e.g., -OH, -COOH, -SH, -NH2) into their substrates by oxidation, reduction and hydrolysis reactions. Cytochrome P450 (CYP) enzymes, which catalyze hydrolysis reactions, play a critical role in defense against inhaled particles. A significant upregulation in the mRNA levels of Phase I CYP enzymes: CYP1B1 and CYP2C8 in females, and CYP2E1, CYP2A6, CYP3A7 and CYP4A14 in males was found upon exposure to CNC materials (Fig. 12). In addition to CYPs a significant over expression of other Phase I enzymes including AOC1 (1.35-folds) and AKR1B10 (1.36-folds). In contrast, a marked downregulation of genes related to several phase I enzymes such as many CYP family members (e.g., CYP2D9, -2B13, -2 J9, -3A7, -4 V2), ADHFE1, FMO5, ALDH1A2, and AKR1C14 was found in females (Fig. 12). The decreased expression of phase I metabolizing enzymes could be due the increased inflammatory responses found in females compared to males. Several studies reported that inflammation and/or infection can lead to downregulation of cytochrome P450s as well as other metabolizing enzymes [71–79]. Based on this, we speculate that the downregulation of phase I enzymes with the exception CYP1B1 and CYP2C8 in females is partially due to the excess production of pro-inflammatory cytokines compared to males (Fig. 5 and Additional file 4: Table S2). In addition to metabolizing xenobiotic compounds and other endogenous metabolites, many CYP enzymes are also involved in catalyzing hydroxylation of estradiol. Importantly, female hormones, such as estrogen, were previously reported to regulate the expression of several CYP isoforms including CYP1B1 and CYP2C8 [80–82]. Both of these CYP isoforms were shown to directly contribute to the oxidative metabolism of estrogen - in particular estradiol metabolism. The unique GO enrichment of “aromatase activity” MF by DEGs in the females (Fig. 11), further highlights the role of female sex hormones in indirectly influencing metabolic processes that could lead to increased oxidative stress in females upon exposure to CNC (Fig. 12).

As opposed to phase I enzymes, phase II enzymes play an important role in protecting the lungs from oxidative injury, either directly or indirectly by inactivating and/or detoxifying xenobiotics and/or foreign substances. While no changes in genes related to phase II enzymes was observed in females upon exposure to CNC, a marked increase in the mRNA levels of several phase II antioxidant/detoxification enzymes and proteins was found in males (Fig. 12). Exposure to CNC in males results in significant upregulation of genes corresponding to NQO1, UGT1A6, NAT8, EPHX1, and GSTA5 (Additional file 1: File S1). Several of these enzymes have been reported to play a role in lowering toxicity associated with exposures to environmental chemicals (e.g., cigarette smoke, exhaust particulates), as well as decreasing the risk of certain types of cancer. Further the upregulation of antioxidant genes, especially GST, could also suggest an adaptive mechanism to remove CYP2E1-derived phase I oxidants in males [83–86]. As certain phase I enzymes can often convert xenobiotics to potent oxidants and oxidizers, the increased expression of CYP1B1 and CYP2C8– influenced by sex hormones – could further lead to higher levels of oxidative stress in females [87, 88]. Thus, it seems likely that accumulation of potentially harmful secondary products (e.g., oxidants/oxidizers) either due to accelerated xenobiotic/cellulose metabolism that is influenced by estrogen or the lack of antioxidant activity and/or detoxification mechanisms could have led to increased oxidative stress responses in females than males (Fig. 12). However, it is crucial to understand whether an acute response or sub-chronic exposure/post-exposure to CNC nanomaterials is required for these gender-based differences to become apparent. Detailed *in vivo* inhalation studies addressing these issues and *in vitro* studies exploring the mechanisms of CNC interactions with different pulmonary cells are almost complete and/or underway. Gender has also been reported to play an important role in incidence and pathogenesis of various lung diseases [89–95]. Epidemiological and experimental data suggest that sex hormones may be important physiological modulators in the lung, and in particular, the role of estrogens in asthma has received considerable attention [89, 91, 96, 97]. Estrogens are synthesized by aromatase, a CYP enzyme located in the endoplasmic reticulum of estrogen producing cells which catalyzes the aromatization of testosterone and androstenedione to 17β-estradiol, the most active estrogen, and estrone [98]. The unique GO enrichment of “aromatase activity” MF identified by the DEGs in only CNC exposed female mice (Fig. 11B) is in line with the role of CYPs in synthesizing estrogens. Estrogens are known to preferentially up-regulate Phase I enzymes, leading to accumulation of toxic metabolites through the bio-activation. Importantly, the mRNA levels of CYP1B1, a known key Phase I enzyme in the metabolism of 17β-estradiol, was significantly elevated only in CNC-exposed female mice along with increased estrogen in the peripheral blood (Additional file 6: Figure S1). The overexpression of CYP1B1 and estrogen in females upon exposure to CNC further supports the estrogen’s role in regulating CYP1B1 via estrogen receptor-α (ERα) [99]. Moreover, the mRNA levels of NCOA3, a member of the steroid receptor coactivator (SRC)/p160 family associated with ERs during transcription, was found to be up-regulated only in CNC exposed female mice. ERs are found on numerous immuno-regulatory cells and estrogen’s actions could divert the immune response toward allergy. Additionally, it has been proposed that estrogen may act directly creating deleterious effects affecting lung mechanics and airway inflammation [93, 96, 100–102]. Several studies suggest that female sex hormones play an important role in inflammatory airway conditions [102], through different but related mechanisms. It has been shown that estrogen promotes a Th2 response, while androgen promotes a Th1 response, which may be relevant in asthma [93, 96, 101, 103–108]. The secretion of cytokines IL-4, IL-5 and IL-13 are central to orchestrating inflammatory responses by Th2 polarized cells. In fact, M2 macrophages and other phagocytic or immune cells in the BAL fluid or airway biopsies of asthmatic patients and/or allergen-challenged mice were shown to secrete a variety of inflammatory factors, including IL-4, IL-5 and IL-13 [101, 105–107]. These included factors that recruit more lymphocytes into the airways, that promote bronchoconstriction and fibrotic tissue remodeling as well as those that enhance collagen deposition for smooth-muscle thickening. A significant increase in IL-5 and IL-13 along with increased recruitment of inflammatory cells (Fig. 3), elevated levels of fibrosis markers (TGF-β1 and collagen) (Fig. 7) and increased airway hyperresponsiveness (Fig. 7c) in females compared to males was also evident in our study. This is further corroborated by previous studies reporting females are more susceptible to the development of severe asthmatic reactions, higher risk of chronic obstructive pulmonary disease and lung cancer [90, 109, 110]. Taken together, these results highlight and suggest the role of CNC materials in potentially triggering Th2 dependent asthmatic responses via a sex-related hormone, estrogen in female mice. Further studies are necessary to explore potential mechanisms involved in estrogen facilitated inflammatory outcomes observed after CNC exposure.

## Conclusion

In conclusion, our findings clearly highlight that pulmonary exposure to respirable CNC materials, leading to inflammation, oxidative stress and other pulmonary responses, were more pronounced in females when compared to male mice. This study also raises the possibility of an increased risk and/or early onset of allergic responses among females upon exposure to CNC materials, mimicking molecules commonly found in microbial cell walls and viral particles such as chitin, polysaccharides and other peptidoglycans. Our study further suggests that the association between nanoparticle exposure and pulmonary inflammatory responses is complex and could be dependent on the type, size and structure of nanoparticles. Furthermore, these responses may be specific to the gender that is being investigated.

## Abbreviations

CNC, cellulose nanocrystals; NC, nanocellulose; CF, cellulose fibers; BAL, bronchial alveolar lavage fluids; AFM, atomic force microscopy; DSL, dynamic light scattering analysis; SEM, scanning electron microscopy; EDX, energy dispersive X-ray spectroscopy; HEPA, high efficiency particulate air; AAALAC, Association for Assessment and Accreditation of Laboratory Animal Care; NIOSH, National Institute of Occupational and Safety Health; OLAW, Office of Laboratory Animal Welfare; HELD, Health Effects Laboratory Division; USP, United States Pharmacopeia; OSHA, Occupational Safety & Health Administration; FPL, Forest Products Laboratory; NaOH, sodium hydroxide; RO, reverse osmosis; LAL, limulus amebocyte lysate; PBS, phosphate buffered saline; AMs, alveolar macrophages; PMNs, polymorphonuclear leukocytes; H_2_O, water; H&E, hematoxylin and eosin; LDH, lactate dehydrogenase; MPO, myeloperoxidase; ELISA, enzyme-linked immunosorbent assay; HNE-His, hydroxynonenal protein adduct; E, estrogen; WBP, whole-body plethysmograph; MCh, methacholine; MIAME, Minimum Information About a Microarray Experiment; RT-qPCR, reverse transcription quantitative polymerase chain reaction; GO, gene ontology; KEGG, Kyoto Encyclopedia of Genes and Genomes; DAVID, Database for Annotation, Visualization and Integrated Discovery; REViGO, Reduce Visualize Gene Ontology; DEGs, differentially expressed genes; ChEA, ChIP Enrichment Analysis; MGCs, multi-nucleated giant cells (MGCs); BP, biological process; MF, molecular function; CC, cellular component; APR, acute phase response; SOD, superoxide dismutase; PEL, permissible exposure limit; TWA, time weighted average; CFR, code of federal regulations; COPD, chronic obstructive pulmonary disease; CYP, cytochrome P450.

## Additional files

Additional file 1: File S1. Differentially expressed mRNA profiles upon pharyngeal aspiration exposure to CNC. The differentially up and down-regulated mRNAs in the lungs of male and female mice 3 months after repeated pharyngeal aspiration with CNC (cumulative dose of 240 μg/mouse). (XLSX 140 kb)

Additional file 2: Table S1. Broad groups of enriched GO_terms based on the GO_slim classification system. The Cellular Component, Biological Process and Molecular Function annotations were classified into broad groups based on the GO-slim classification system using CateGOrizer. (XLSX 18 kb)

Additional file 3: File S2. Enriched gene ontology terms associated with differentially expressed mRNAs in female and male mice upon exposure to CNC. The enriched Cellular Component (CC), Biological Process (BP) and Molecular Function (MF) annotations associated with the differentially expressed genes were predicted using Database for Annotation, Visualization and Integrated Discovery (DAVID) software tool. (XLSX 68 kb)

Additional file 4: Table S2. KEGG pathway enrichment analysis of up and down-regulated genes in the lungs of male and female mice exposed to CNC. (XLSX 14 kb)

Additional file 5: File S3. The list of key transcriptional factors which regulated the expression of the upregulated differentially expressed genes in males and females upon exposure to CNC. The prediction of transcription factors was performed using ChEA (ChIP Enrichment Analysis) software tool. A P value cutoff of ≤ 0.05 was considered significant in each case. (XLSX 32 kb)

Additional file 6: Figure S1. Levels of estrogen measured in the serum of female mice 3 month post repeated exposure with CNC (cumulative dose of 240 μg/mouse). Mean ± SEM (*n* = 10 mice/group). *p < 0.05, vs control PBS-exposed mice. (PDF 301 kb)
