# Supplementary figures and images for: Gender differences in murine pulmonary responses elicited by cellulose nanocrystals
Source: Part Fibre Toxicol. 2016 Jun 8;13:28. doi: 10.1186/s12989-016-0140-x (PMC4898310; doi:10.1186/s12989-016-0140-x)

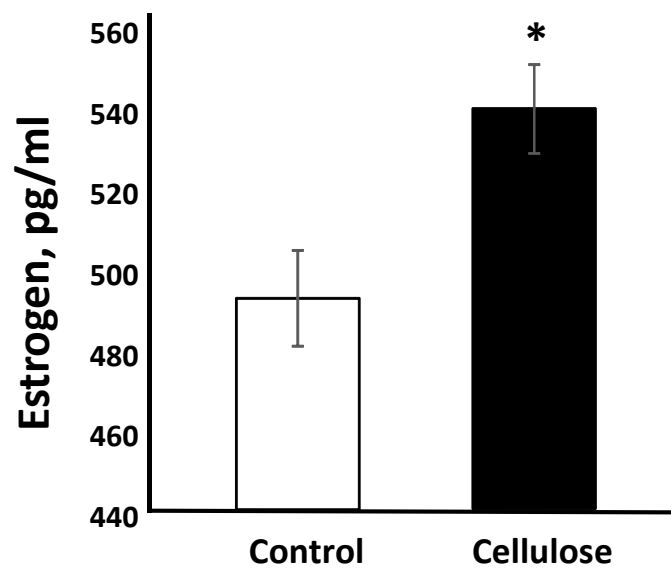

Fig.S1.

Supplement: Supplementary file 6 — Levels of estrogen measured in the serum of female mice 3 month post repeated exposure with CNC (cumulative dose of 240 μg/mouse). Mean ± SEM (n = 10 mice/group). *p < 0.05, vs control PBS-exposed mice. (PDF 301 kb) [file 12989_2016_140_MOESM6_ESM.pdf]
